# Effect of the Measurement Interface on Maximal Expiratory Pressure During Voluntary Cough in Young Adults

**DOI:** 10.3390/diagnostics16213626

**Published:** 2026-11-03

**Authors:** Rodrigo Muñoz-Cofré, Cristian Villar Gonzalez, Francisca Yáñez Quiñones, Paul Medina-González, Mariano del Sol, Raúl Escobar Henríquez, Máximo Escobar-Cabello

**Affiliations:** 1 Postdoctorado en Ciencias Morfológicas, Universidad de La Frontera, Temuco 4811230, Chile; 2 Escuele de Kinesiología, Universidad Católica del Maule, Talca 4811230, Chile; cristian.villar.gonzalez@outlook.com (C.V.G.); francisca.y.q@outlook.com (F.Y.Q.); pmedina@ucm.cl (P.M.-G.); maxfescobar@gmail.com (M.E.-C.); 3 Centro de Excelencia en Estudios Morfológicos y Quirúrgicos, Universidad de La Frontera, Temuco 4811230, Chile; mariano.delsol@ufrontera.cl; 4 Departamento de Neurología Pediátrica, Escuela de Medicina, Pontificia Universidad Católica de Chile, Santiago 8320165, Chile; rescobah@uc.cl

**Keywords:** cough, maximal espiratory pressure, oronasal mask, nozzle

## Abstract

**Background/Objectives**: To determine whether there are differences between a nozzle and an oronasal mask in the measurement of maximal expiratory pressure (MEP) during a voluntary cough maneuver in young university students. **Methods:** Forty university students aged 20–25 years were recruited, comprising 20 males and 20 females. Forced vital capacity and MEP were measured during a voluntary cough maneuver. All participants were assessed using both interfaces. The order of the interfaces used to measure MEP during the voluntary cough maneuver was randomized, once the order was determined, the evaluations were conducted on two separate days: one for the nozzle and another for the oronasal mask. Subsequently, participants were asked to report their perception of air leakage and comfort associated with each device. **Results:** There was no significant main effect of the interface on MEP after adjusting for gender, peak expiratory flow (PEF), and body mass index (BMI) (F(1.36) = 3.285; *p* = 0.078; η^2^p = 0.084). There was a significant interaction between interface and BMI (F(1,36) = 6.391; *p* = 0.016; η^2^p = 0.151). Gender and PEF were significantly associated with MEP (gender: F(1,36) = 16.698; *p* < 0.001; η^2^p = 0.317; PEF: F(1,36) = 8.985; *p* = 0.005; η^2^p = 0.200). **Conclusions:** In young adults, the measurement interface alone did not significantly alter MEP. The relationship between BMI and MEP varied according to the interface used, as evidenced by the interface × BMI interaction; gender and PEF were significantly associated with MEP.

## 1. Introduction

Functionally, cough is a protective airway-clearance mechanism [1,2,3]. It is triggered to clear sputum and bronchial secretions and plays a key role in protecting the lower airways from the aspiration of foreign material [3,4,5]. Although cough is defined as a reflex, it can also be voluntarily initiated, making it an important therapeutic tool for maintaining airway patency [6,7]. In this context, cough monitoring is crucial, particularly in pathological cases. Consequently, efforts are currently being directed toward the accurate measurement of coughing and the potential diagnosis of disorders affecting any of its phases—steps that would lead to changes in or additions to the therapeutic approach [8,9].

For physiotherapists, coughing is one of the most widely used strategies to support mucociliary clearance, and consequently, airway patency. In this context, cough has become part of the clinical scores aimed at diagnosing respiratory disorders [10,11,12].

Cough is influenced by multiple interacting components, including inspiratory volume, glottic function, expiratory muscle force generation, and expiratory airflow. However, inadequate respiratory muscle strength may result in lower expiratory pressures, thereby impairing the cough mechanism [13]. Therefore, the accurate assessment of respiratory muscle strength may have a direct impact on subsequent therapeutic interventions.

In pulmonary function laboratories, several indirect methods are available to assess and/or measure cough strength, including peak cough flow measured using a peak flow meter, gastric pressure during the voluntary cough maneuver, maximal insufflation capacity, and, most commonly, maximal expiratory pressure (MEP). MEP has been described as a sensitive and appropriate indicator of cough strength [14]. Furthermore, it is considered a readily accessible test that allows the overall assessment of expiratory muscle strength [15,16]. On the other hand, evaluating the voluntary cough maneuver in a young, apparently healthy population would make it possible to minimize the potential influence of age-related respiratory, neuromuscular, or pulmonary changes, thereby determining the effect of the interface under relatively homogeneous physiological conditions.

Traditionally, MEP is measured using a nozzle connected to an aneroid manometer or a plethysmograph [16]. However, the oronasal mask has emerged as a potential alternative interface for this assessment. Previous studies have reported differences in air leakage between these interfaces, with less leakage observed when using a nozzle [17,18,19]. Nevertheless, available evidence has focused primarily on respiratory pressure measurements or air leakage, and there is limited evidence regarding whether the measurement interface influences the MEP values obtained during a voluntary cough maneuver. In particular, it remains unclear whether an oronasal mask—by allowing the participant to perform the cough maneuver without placing a nozzle in the mouth—might alter the pressure generated during the maneuver. This represents a significant methodological gap, as the interface used during assessment could influence the magnitude and reproducibility of MEP measurements.

Based on this gap in knowledge, we hypothesized that the use of an oronasal mask would yield higher MEP values during a voluntary cough maneuver compared to the use of a nozzle, as the mask might allow for a more natural cough pattern and reduce potential interference with the oral phase of the maneuver. Therefore, the aim of this study was to determine whether MEP values obtained during a voluntary cough maneuver differ according to the measurement interface used (nozzle versus oronasal mask) in young university students.

## 2. Materials and Methods

We conducted a longitudinal repeated-measures study at the Pulmonary Function Laboratory of the Department of Kinesiology, Universidad Católica del Maule, Talca, Chile, in collaboration with the Neuromuscular Diseases Program of the Neurodevelopment Unit, Hospital Clínico de la Pontificia Universidad Católica de Chile. MEP was measured during a voluntary cough maneuver by a certified evaluator (certified by the Ministry of Health, Santiago, Chile) using a body plethysmograph (Platinum Elite DL^®^, St. Paul, MN, USA). This plethysmograph features an integrated respiratory pressure measurement module; it is important to highlight that it is considered the gold standard in the field of respiratory function assessment. The MEP obtained during a voluntary cough maneuver was compared using a nozzle and an oronasal mask (Figure 1). To assess the subjective conditions associated with the procedure, a subjective perception questionnaire was administered. The primary study variable was determined to be MEP resulting from the voluntary cough maneuver performed with the nozzle and the oronasal mask. Secondary measures included the perception of leaks and the comfort of the interfaces.

### 2.1. Participants

The target population consisted of 4839 undergraduate students enrolled at Universidad Católica del Maule in 2025. The required sample size was estimated using the standard finite population formula, assuming maximum heterogeneity (50%), a 15% margin of error, and a 95% confidence level. With these parameters, the calculated minimum sample size was 42 participants. Due to the availability and eligibility of participants during the recruitment period, the final sample consisted of 40 undergraduate students, including 20 males and 20 females. All participants were required to read and sign an informed consent form before enrollment. The inclusion criteria were: (i) participants in the 20-to-25 age range; (ii) forced expiratory volume in the first second (FEV_1_) and forced expiratory flow at 25–75% of forced vital capacity (FEF_25–75_) ≥80% of the predicted value; and (iii) body mass index (BMI) between 18.5 and 29.9 kg/m^2^. Participants were excluded if they: (i) had structural abnormalities of the thoracic cage; (ii) were a current smoker, former smoker, and occasional smoker; (iii) acute respiratory infections; (iv) chronic respiratory disease; or (v) difficulty understanding and/or performing the maneuver. The study was approved by the Scientific Ethics Committee of Universidad Católica del Maule (Resolution Act No. 23/2025). The recruitment process took place between March and June 2025, beginning with a presentation of the research at the Student Health Department of the Universidad Católica del Maule. Additionally, student councils from the university’s academic programs were contacted; presentations were then held for those programs that expressed an interest in participating.

### 2.2. Measurements

#### 2.2.1. Forced Vital Capacity

Briefly, participants placed the pneumotachograph in their mouth and breathed at tidal volume for five respiratory cycles. They were then instructed to perform a maximal inspiration followed by a maximal forced expiration. A nose clip was used, and standardized verbal encouragement was provided in accordance with current regulations. The best maneuver from a minimum of three acceptable and reproducible attempts was selected according to current guidelines (ATS/ERS, 2019) [20].

#### 2.2.2. Measurement of Maximal Expiratory Pressure

All participants were assessed using both interfaces. First, the order of the interfaces used to measure MEP during the voluntary cough maneuver—nozzle or oronasal mask—was randomized using the online Research Randomizer software, and there was a 24-h interval between the evaluations of each interface. Second, participants were placed in a seated position, and the corresponding interface was positioned: the nozzle was placed in the mouth, whereas the oronasal mask was positioned over the nose and mouth (Figure 2). Third, MEP was measured during the voluntary cough maneuver. Participants were instructed to breathe at tidal volume for five respiratory cycles. They were subsequently instructed to perform a maximal inspiration. The pneumotachograph was then occluded, after which participants were instructed to perform a cough effort, a one-minute rest was allowed between each attempt. Prior to the start of the evaluations, each participant was given the opportunity to make a practice attempt using the corresponding interface, with the aim of clarifying any questions and reinforcing the measurement protocol. The best maneuver from a minimum of three acceptable and reproducible attempts was selected according to current guidelines (ATS/ERS, 2002) [16]. A maneuver was defined as acceptable when the participant performed it in a technically adequate manner according to the specified protocol: seated position, inspiration to total lung capacity (TLC), maximal expiratory effort, correct interface positioning, and execution of the voluntary cough maneuver. Regarding reproducibility, once the acceptability criterion was met, the highest value was recorded from the three voluntary cough maneuvers showing less than 5% variability among them [16].

#### 2.2.3. Interfaces

(i) Nozzle (MedGraphics Elite Platinum plethysmograph; rubber, 25 mm in diameter and 50 mm long, with an 80 mm oral seal at the proximal end—to which the participant connected—and a distal end connected to the plethysmograph’s pneumotachograph); and (ii) oronasal mask (Hans Rudolph 6450 Series V2, size No. 5/Large), made of medical-grade silicone and featuring an anatomically contoured face seal, an integrated chin cup, and a five-point headgear attachment system. The mask was connected to the plethysmograph’s pneumotachograph.

#### 2.2.4. Perception of Air Leakage and Comfort

To assess air leakage and comfort during the procedure, the following methods were used. To determine perceived air leakage, participants were asked directly: “Did you perceive air leakage with the nozzle (yes/no)? Responses were recorded dichotomously (yes/no). To assess perceived comfort, a five-point Likert-type scale was used with the following response options: Too uncomfortable, Uncomfortable, Somewhat uncomfortable, Comfortable, and Very comfortable. Finally, it is important to note that this scale was developed specifically for this study and has not undergone formal validation.

### 2.3. Statistical Analysis

Statistical analyses were performed using IBM SPSS Statistics version 25. Continuous variables were expressed as mean ± standard deviation (SD), and categorical variables as absolute and relative frequencies. The distribution of continuous variables was assessed using the Shapiro–Wilk test. To compare baseline variables between genders, the Student’s *t*-test or the Mann–Whitney U test was used, depending on the data distribution. To evaluate the effect of measurement interface on MEP during the voluntary cough maneuver, a repeated-measures analysis of covariance (ANCOVA) was performed, with measurement interface (nozzle vs. oronasal mask) included as the within-subject factor, gender as the between-subject factor, and peak expiratory flow (PEF) and body mass index (BMI) as covariates. Main effects of interface, gender, PEF, and BMI, as well as the interaction terms interface × gender, interface × PEF, and interface × BMI were evaluated. Because the within-subject factor consisted of two levels, the sphericity assumption was not applicable. Estimated marginal means with 95% confidence intervals were obtained from the ANCOVA model. Partial eta squared (η^2^p) was used as a measure of effect size, with values of 0.01, 0.06, and 0.14 interpreted as small, medium, and large effects, respectively. To further characterize the significant interface × BMI interaction, separate multiple linear regression models were performed for each measurement interface, with MEP as the dependent variable and gender, PEF, and BMI as independent variables. For secondary analyses of perceived air leakage, paired categorical responses between interfaces were compared using McNemar’s test. For the analysis of interface comfort, data are presented as medians and percentages; additionally, for statistical analysis, either a paired-sample Student’s *t*-test or a Wilcoxon test was used, depending on the data distribution. All statistical tests were two-sided, and statistical significance was set at *p* < 0.05.

## 3. Results

A total of 40 participants completed the study protocol, including 20 males and 20 females. Anthropometric and pulmonary function characteristics are presented in Table 1. Males had greater body weight, height, BMI, forced vital capacity (FVC), volume forced expiratory in the first second (FEV_1_), forced expiratory flow between 25% and 75% of vital capacity (FEF_25–75_), and PEF values than females, whereas no significant difference was observed in age. the values from the descriptive analysis of MEP, measured during the voluntary cough maneuver, were 82.58 ± 40.83 versus 112.20 ± 38.20 cmH_2_O (95% confidence interval = −41.05 to −18.10) for the nozzle and oronasal mask, respectively. For males, the values obtained with the nozzle and oronasal mask were 97.67 ± 40.50 versus 129.90 ± 40.18 cmH_2_O (95% confidence interval = −45.84 to −18.60), respectively. In females, the corresponding values were 69.00 ± 37.10 and 96.20 ± 28.83 cmH_2_O (95% confidence interval = −48.44 to −5.963) for the nozzle and oronasal mask, respectively.

A repeated-measures ANCOVA was performed to examine the effect of measurement interface on MEP, with gender included as a between-subject factor and PEF and BMI as covariates. The main effect of interface was not statistically significant, F(1,36) = 3.285, *p* = 0.078, partial η^2^ = 0.084. A significant main effect of gender was observed, F(1,36) = 16.698, *p* < 0.001, partial η^2^ = 0.317, whereas the main effects of PEF and BMI were F(1,36) = 8.985, *p* = 0.005, partial η^2^ = 0.200, and F(1,36) = 0.286, *p* = 0.596, partial η^2^ = 0.008, respectively (Table 2).

No significant interaction was observed between interface and gender, F(1,36) = 0.010, *p* = 0.920, partial η^2^ < 0.001, or between interface and PEF, F(1,36) = 0.581, *p* = 0.451, partial η^2^ = 0.016. In contrast, a significant interface × BMI interaction was observed, F(1,36) = 6.391, *p* = 0.016, partial η^2^ = 0.151, indicating that the association between BMI and MEP differed according to the measurement interface (Table 2). The adjusted estimated marginal means were 125.575 for the oronasal mask interface and 97.475 for the nozzle interface, after accounting for gender, PEF, and BMI. The adjusted between-interface mean difference was 28.100 (95% CI: 17.378–38.822; *p* < 0.001).

To further characterize the significant interface × BMI interaction, separate multiple linear regression models were performed for each interface, including PEF, gender, and BMI as predictors. The model for the nozzle was statistically significant, F(3,36) = 12.244, *p* < 0.001, R^2^ = 0.505, adjusted R^2^ = 0.464. PEF was positively associated with MEP (B = 8.927, β = 0.403, *p* = 0.010), while gender was also significantly associated with MEP (B = −42.098, β = −0.463, *p* = 0.003). The association between BMI and MEP was negative but did not reach statistical significance (B = −4.775, β = −0.215, *p* = 0.104) (Table 3).

The model for the oronasal mask was also statistically significant, F(3,36) = 17.136, *p* < 0.001, R^2^ = 0.588, adjusted R^2^ = 0.554. PEF was positively associated with MEP (B = 6.443, β = 0.316, *p* = 0.026), and gender was significantly associated with MEP (B = −40.789, β = −0.487, *p* = 0.001). BMI was positively associated with MEP, although this association was not statistically significant (B = 2.389, β = 0.117, *p* = 0.327). Thus, the BMI slopes were opposite in direction for the two interfaces (Table 3).

The McNemar test revealed statistically significant differences in air leakage between the oronasal mask and nozzle among females, but not among males. Among females (*n* = 20), 18 participants reported air leakage with the oronasal mask but not with the nozzle, whereas 2 participants reported the opposite pattern. This difference was statistically significant (two-sided exact McNemar test, *p* = 0.0004). Among males (*n* = 20), 12 participants reported air leakage with the oronasal mask but not with the nozzle, whereas 8 participants reported the opposite pattern; this difference was not statistically significant (*p* = 0.503). These findings indicate that air leakage differed significantly between the two interfaces among females, whereas no statistically significant difference was observed among males (Table 4).

Comfort ratings differed significantly between the two interfaces (two-sided Wilcoxon signed-rank test, W = 60.5, *p* = 0.003). The median rating was 1 (comfortable) for the oronasal mask and 2 (somewhat uncomfortable) for the nozzle, indicating greater perceived comfort with the oronasal mask. A more favorable comfort rating was reported for the oronasal mask by 21 participants, whereas 5 participants reported a more favorable rating for the nozzle and 14 reported identical ratings for both interfaces. The complete distribution of responses across all five categories is presented in Table 5.

## 4. Discussion

The aim of this study was to determine whether differences exist between a nozzle and an oronasal mask in the measurement of MEP during voluntary cough maneuver. The main findings were as follows: (i) the measurement interface did not show a statistically significant main effect on MEP after adjusting for gender, PEF, and BMI; (ii) a significant interaction was observed between the interface and BMI; and (iii) gender and PEF showed a significant association with MEP. The significant interaction between the interface and BMI suggests that the relationship between body composition and expiratory pressure could vary depending on the interface used for measurement. Notably, no significant interaction was observed between the interface and gender or between the interface and PEF, indicating that the association of the measurement interface with MEP was not significantly modified by these variables.

One notable result was the significant interaction between the interface and BMI (*p* = 0.016, partial η^2^ = 0.151), indicating that the association between BMI and MEP differed depending on the measurement interface (Table 2). There is limited information linking MEP measurement during a voluntary cough maneuver to the specific interface used. However, Singh et al. evaluated the efficacy of nasal mask ventilation versus standard oronasal mask ventilation in anesthetized obese patients with a BMI >25 kg/m^2^. Their main conclusion was that nasal mask ventilation was more effective than conventional oronasal mask ventilation in anesthetized obese patients. Facial and body characteristics can influence leakage and interface seal efficacy; individuals with higher BMI may possess characteristics that promote greater air leakage with oronasal masks [21]. Although our assessment involved measuring MEP during a voluntary cough maneuver—implying different respiratory mechanics—it is worth highlighting methodological aspects that might explain this relationship: (i) the motor pattern of the cough, as an oronasal mask might allow for a more natural cough than a nozzle, which is placed entirely inside the mouth; and (ii) the interface and applied pressure, given that the oronasal mask was equipped with a silicone seal and a headgear strap system—features that could have enhanced interface efficacy regardless of the participants’ facial characteristics.

Another result obtained was the significant association between gender, PEF, and the MEP resulting from a voluntary cough maneuver (Table 3); thus, both were predictors/associated factors, and there was no interaction between interface and gender, nor between interface and PEF. This means that their association with MEP did not depend significantly on the interface. Gender-related differences have been documented regarding airway lumen area, lung size, thoracic configuration, and muscle strength [22,23,24,25]. Specifically, Lista-Paz et al. (2023) reported MEP values of 142 cmH_2_O in females and 195 cmH_2_O in males [26]. Similarly, Khanduri et al. observed significantly higher MEP values in males (86.37 ± 26.96 cmH_2_O) than in females (56.04 ± 18.29 cmH_2_O; *p* = 0.001) [27]. This is particularly relevant considering that forced expiration relies on the abdominal and internal intercostal muscles. Therefore, when these muscles function near their optimal force-generating capacity, PEF can increase, and consequently, cough strength may improve [28,29,30]. These arguments are consistent with the association between PEF and gender observed in the results of the present study (Table 3).

Another finding was the presence of air leaks, which was significantly greater with the oronasal mask than with the nozzle in the total sample and among females. In this regard, Fiore et al. (2004) determined that assessing MEP using an oronasal mask resulted in air leaks in 60% of the patients evaluated [18]. The leaks occurred predominantly at the base of the nose and persisted even after optimizing the fit of the oronasal mask to the patient’s face [15,17]. These findings are consistent with those observed among the female participants in the present study, where 18 individuals (McNemar = 12.80; *p* = 0.0004) reported air leaks when using the oronasal mask. This finding highlights the need to further optimize this interface, particularly given that it yielded the descriptively higher MEP values. In this context, the next step would be to improve the securing mechanisms in order to come as close as possible to the true maximal effort.

No studies were identified that analyzed participants’ subjective perceptions regarding the assessment of MEP in relation to the interface used. This is an important consideration because such information provides subjective feedback on the measurement procedure, allowing for the identification of potential limitations and/or advantages associated with each interface. In the present study, participants were asked to rate the comfort of the nozzle and the oronasal mask. Both males and females rated the use of the oronasal mask as “comfortable.” In contrast, males rated the nozzle as “somewhat uncomfortable”, whereas females—consistent with their perception of the oronasal mask—rated it as “comfortable”.

The limitations of this study include: (i) the lack of an instrument to objectively quantify pressure leakage from any of the devices; (ii) despite the external securing mechanism for the oronasal mask, participants perceived air leaks due to the high positive pressure generated during measurement, indicating a need for further research to improve the mask support system; and (iii) the relatively small sample size. Future studies should consider extending the research to other age groups across the lifespan, as well as to individuals with respiratory and neuromuscular diseases, in whom expiratory muscle strength is reduced.

## 5. Conclusions

In young adults, the descriptive analysis showed a higher MEP value when using the oronasal mask compared to the nozzle during the voluntary cough maneuver. The analysis adjusted for the covariates of gender, PEF, and BMI, and the measurement interface did not show a statistically significant main effect on PEM during a voluntary cough maneuver. However, the significant interaction between the measurement interface and BMI suggests that the relationship between BMI and MEP might vary depending on the interface used. Gender and PEF were significantly associated with PEM, whereas no significant interactions were observed between the interface and gender or between the interface and PEF. These findings highlight the importance of considering interface characteristics, body composition, and participant-related factors when assessing MEP during voluntary cough maneuvers. Future studies incorporating objective measurements of interface leaks and larger, more diverse samples are needed to clarify the influence of the measurement interface on MEP.

## Figures and Tables

**Figure 1:**
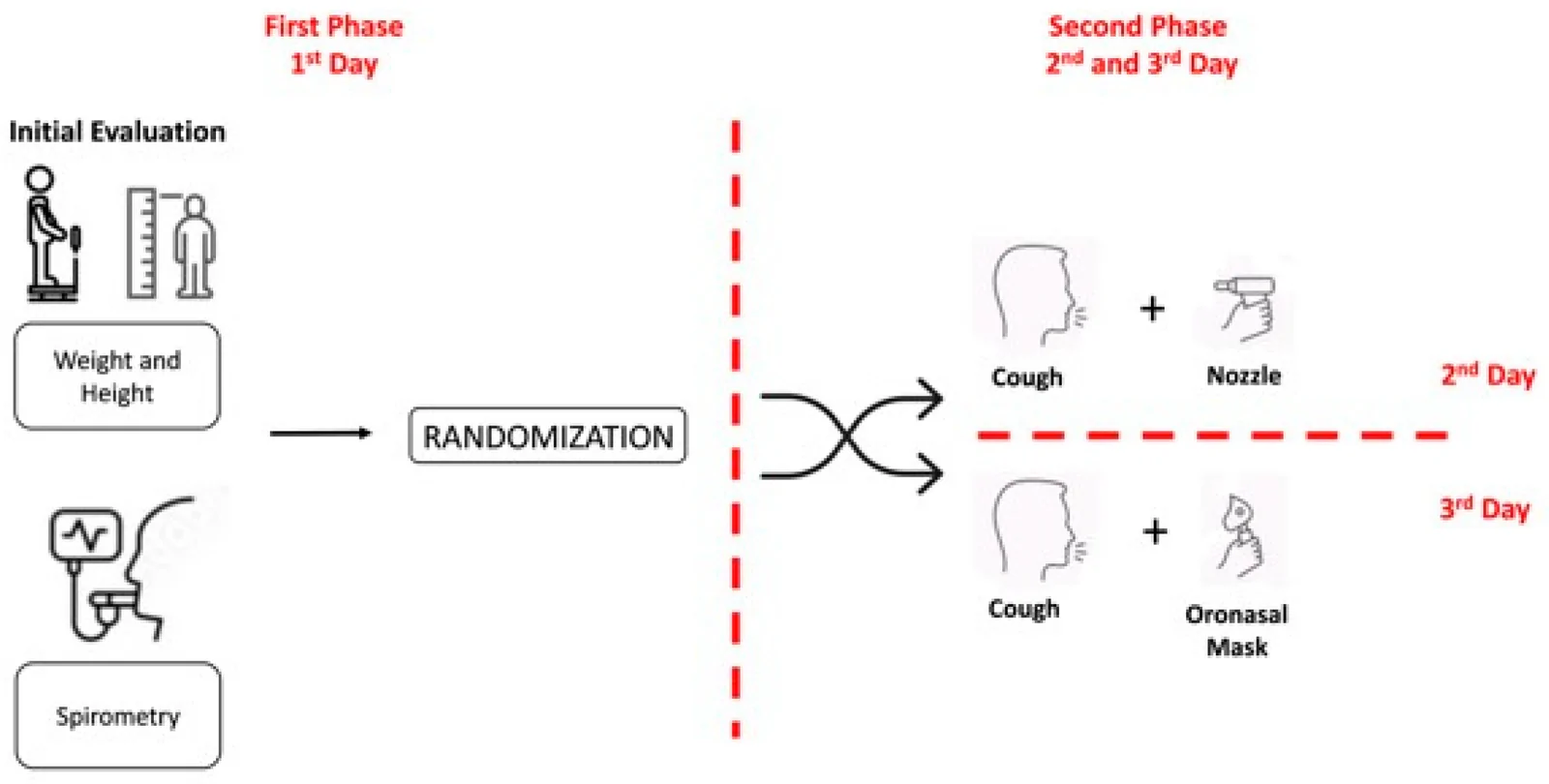
Flowchart.

**Figure 2:**
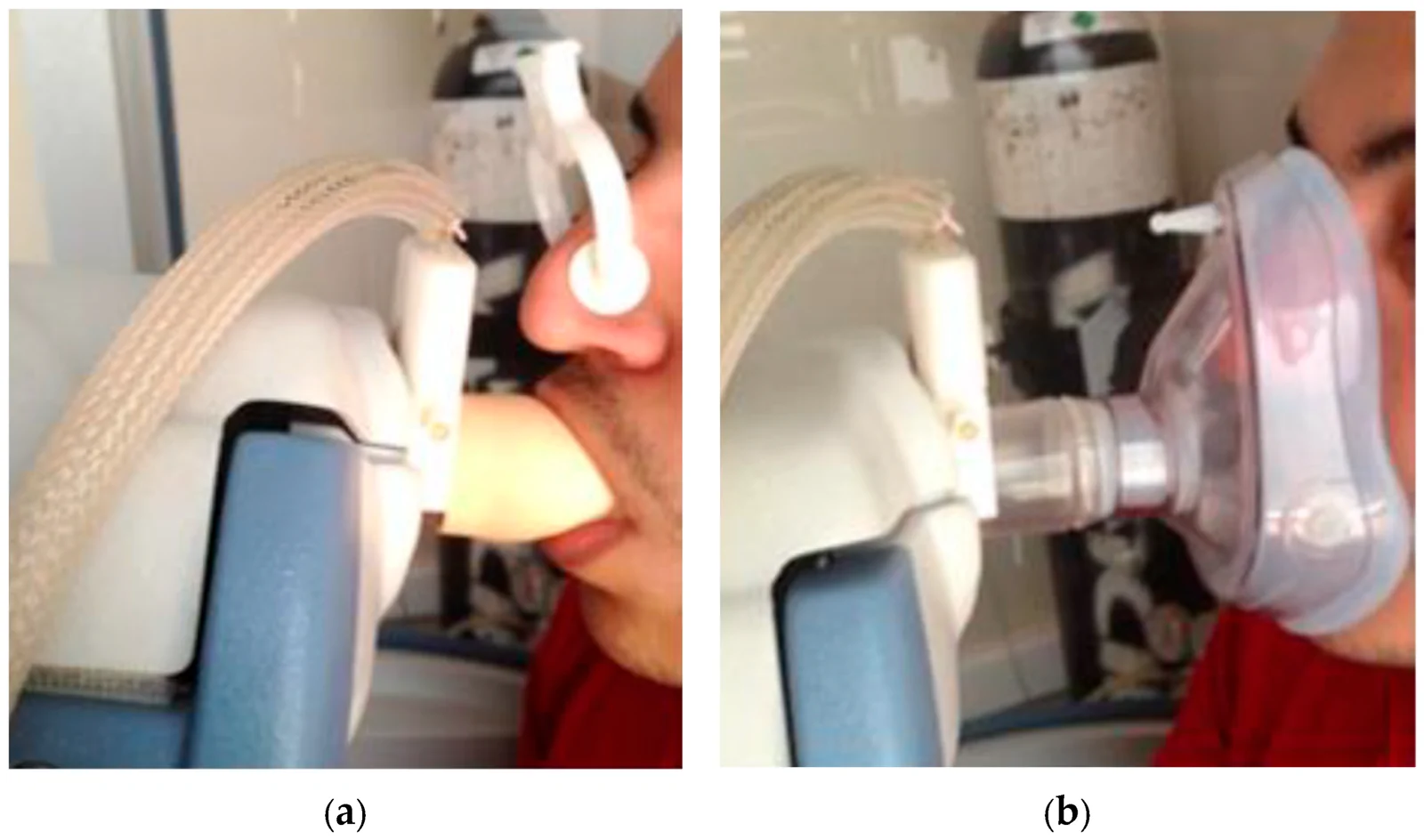
Interfaces used for MEP measurement during a voluntary cough maneuver. (**a**) Nozzle. (**b**) Oronasal mask.

**Table 1:** Anthropometric characteristics and pulmonary function of the study sample.

	Total Sample (*n*:40)	Males (*n*:20)	Females (*n*:20)	Valor *p*
Age (years)	22 ± 1	23 ± 2	22 ± 2	0.865
Weight (kg)	63.10 ± 10.30	70.22 ± 7.01	55.53 ± 7.95	0.0001 ^a^
Height (m)	1.68 ± 0.09	1.76 ± 0.04	1.61 ± 0.06	0.0001 ^a^
BMI (kg/m^2^)	22.14 ± 2.07	22.73 ± 0.2.07	21.36 ± 1.9	0.0369 ^b^
FVC (L)	4.60 ± 0.97	5.37 ± 0.59	3.83 ± 0.57	0.0001 ^a^
FVC (L) % Pred	105.40 ± 11.61	104.8 ± 10.81	106.1 ± 12.61	-
FEV_1_ (L)	3.89 ± 0.71	4.42 ± 0.53	3.35 ± 0.40	0.0001 ^b^
FEV_1_ (L) % Pred	103.80 ± 11.47	100.5 ± 10.59	107.0 ± 12.04	-
FEF_25–75_ (L/s)	4.17 ± 0.98	4.45 ± 0.98	3.90 ± 0.92	0.0321 ^b^
FEF_25–75_ (L/s) % Pred	97.63 ± 23.29	90.65 ± 18.80	104.6 ± 25.64	-
PEF (L/s)	8.11 ± 2.08	9.27 ± 2.25	6.94 ± 0.97	0.0001 ^b^
PEF (L/s) % Pred	101.91 ± 19.83	98.90 ± 23.21	104.9 ± 15.80	-

FVC: forced vital capacity; FEV_1_: forced expiratory volume in the first second; FEV_1_/FVC: ratio of forced expiratory volume in the first second to forced vital capacity; %: percentage; FEF_25–75_: forced expiratory flow between 25% and 75% of forced vital capacity; PEF: peak expiratory flow; L/s: liters per second; a: Mann–Whitney U test; b: Student’s *t*-test. Since baseline variables were compared between sexes, Student’s *t*-test or the Mann–Whitney U test was used.

**Table 2:** Repeated-measures ANCOVA examining the effects of measurement interface, sex, PEF, and BMI on MEP.

Effect	F	df	*p*-Value	Partial η^2^
Interface	3.285	1,36	0.078	0.084
Gender	16.698	1,36	<0.001	0.317
PEF	8.985	1,36	0.005	0.200
BMI	0.286	1,36	0.596	0.008
Interface × Gender	0.010	1,36	0.920	<0.001
Interface × PEF	0.581	1,36	0.451	0.016
Interface × BMI	6.391	1,36	0.016	0.151

MEP: maximal expiratory pressure; PEF: peak expiratory flow; BMI: body mass index. Interface was entered as the within-subject factor, gender as the between-subject factor, and PEF and BMI as covariates. Partial η^2^ represents partial eta squared.

**Table 3:** Multiple linear regression for improvement in pressure espiratory maximal.

Predictor	Nozzle B (β)	*p*-Value	Oronasal Mask B (β)	*p*-Value
PEF	8.927 (0.403)	0.010	6.443 (0.316)	0.026
Gender	−42.098 (−0.463)	0.003	−40.789 (−0.487)	0.001
BMI	−4.775 (−0.215)	0.104	2.389 (0.117)	0.327
Constant	151.805	0.023	40.813	0.452
R^2^	0.505		0.588	
Adjusted R^2^	0.464		0.554	
F	12.244	<0.001	17.136	<0.001

B: unstandardized regression coefficient; β: standardized regression coefficient; PEF: peak expiratory flow; BMI: body mass index. Gender was coded according to the study database.

**Table 4:** Perceived Air Leakage According to Measurement Interface.

Females						
Air leakage with Nozzle		Air leakage with Oronasal mask				
		Yes	No	Total	McNemar	*p* value (two-sided)
	Yes	0	2	2	12.80	0.0004
	No	18	0	18		
	Total	18	2	20		
Males						
Air leakage with Nozzle		Air leakage with Oronasal mask				
		Yes	No	Total	McNemar	*p* value (two-sided)
	Yes	0	8	8	0.80	0.503
	No	12	0	12		
	Total	12	8	20		

Values represent participants who reported perceived air leakage. McNemar’s test was used for paired comparisons between interfaces.

**Table 5:** Perception of comfort when using the interface.

Comfort Rating	Oronasal Mask, *n* (%)	Nozzle, *n* (%)
0—Very comfortable	5 (12.5%)	2 (5.0%)
1—Comfortable	22 (55.0%)	12 (30.0%)
2—Somewhat uncomfortable	11 (27.5%)	15 (37.5%)
3—Uncomfortable	2 (5.0%)	7 (17.5%)
4—Extremely uncomfortable	0 (0.0%)	4 (10.0%)
Total	40 (100%)	40 (100%)

## Data Availability

The data presented in this study are available on request from the corresponding author due to privacy restrictions.

## Abbreviations

The following abbreviations are used in this manuscript:

MEP	Maximal Espiratory Pressure
PEF	Peak Espiratory Flow
